# Adenosine A_2A_ Receptors Modulate Acute Injury and Neuroinflammation in Brain Ischemia

**DOI:** 10.1155/2014/805198

**Published:** 2014-08-05

**Authors:** Felicita Pedata, Anna Maria Pugliese, Elisabetta Coppi, Ilaria Dettori, Giovanna Maraula, Lucrezia Cellai, Alessia Melani

**Affiliations:** ^1^Department of Neuroscience, Psychology, Drug Research and Child Health (NEUROFARBA), University of Florence, Viale Pieraccini 6, 50139 Florence, Italy; ^2^Department of Health Sciences, University of Florence, Viale Pieraccini 6, 50139 Florence, Italy

## Abstract

The extracellular concentration of adenosine in the brain increases dramatically during ischemia. Adenosine A_2A_ receptor is expressed in neurons and glial cells and in inflammatory cells (lymphocytes and granulocytes). Recently, adenosine A_2A_ receptor emerged as a potential therapeutic attractive target in ischemia. Ischemia is a multifactorial pathology characterized by different events evolving in the time. After ischemia the early massive increase of extracellular glutamate is followed by activation of resident immune cells, that is, microglia, and production or activation of inflammation mediators. Proinflammatory cytokines, which upregulate cell adhesion molecules, exert an important role in promoting recruitment of leukocytes that in turn promote expansion of the inflammatory response in ischemic tissue. Protracted neuroinflammation is now recognized as the predominant mechanism of secondary brain injury progression. A_2A_ receptors present on central cells and on blood cells account for important effects depending on the time-related evolution of the pathological condition. Evidence suggests that A_2A_ receptor antagonists provide early protection via centrally mediated control of excessive excitotoxicity, while A_2A_ receptor agonists provide protracted protection by controlling massive blood cell infiltration in the hours and days after ischemia. Focus on inflammatory responses provides for adenosine A_2A_ receptor agonists a wide therapeutic time-window of hours and even days after stroke.

## 1. Introduction

Ischemic stroke is the second leading cause of death in major industrialized countries, with a mortality rate of around 30%, and the major cause of long-lasting disabilities [1]. Ischemic stroke results from a transient or permanent reduction in cerebral blood flow which is, in most cases, caused by the occlusion of a major brain artery, either by an embolus or by local thrombosis. Currently, there is no promising pharmacotherapy for acute ischemic stroke aside from intravenous or intra-arterial thrombolysis. Yet, because of the narrow therapeutic time-window involved, thrombolytic application is very restricted in clinical settings [2]. Neuroprotective drugs such as glutamate receptor antagonists have shown therapeutic potential in animal stroke trials but have failed to be efficacious during clinical trials [3, 4]. Death-signaling proteins involved in the progression from N-methyl-D-aspartic acid (NMDA) receptor stimulation to excitotoxic neuronal death emerged as possible novel targets for neuroprotection. In particular, inhibition of activation of transcription factors and related proteins, including p38, JNK, and SREBP1, is neuroprotective in animal models of stroke [5]. On the other hand, ischemia is a multifactorial pathology characterized by different events evolving in the time. After ischemia the early massive increase of extracellular glutamate is followed by activation of resident immune cells, that is, microglia, and production or activation of inflammation mediators [6]. Proinflammatory cytokines, which upregulate cell adhesion molecules, exert an important role in promoting neutrophil infiltration and accumulation in brain parenchyma [7, 8]. Although after ischemia precocious activation of immune cells may be neuroprotective and supportive for regeneration, protracted neuroinflammation is now recognized as the predominant mechanism of secondary brain injury progression.

The extracellular adenosine concentration increases dramatically during* in vivo* ischemia as demonstrated first by the cortical cup technique [9, 10] and later on by the microdialysis technique [11–15]. The increase of adenosine extracellular level is attributable to different reasons. Early after ischemia, the increase of adenosine is mainly attributable to extracellularly released ATP [16] that is hydrolysed by ectonucleotidases (NTPDases 1, 2, and 3 that convert ATP to ADP and AMP) and ecto-5′-nucleotidase that converts AMP to adenosine [17, 18]. Thereafter adenosine* per se* is mainly released from cells likely by the equilibrative nucleoside transporter (ENT) 2 [16]. Inhibition of adenosine-uptake processes due to downregulation of concentrative nucleoside transporters (CNT) 2 and 3 and of the ENT1 also contributes to the extracellular adenosine increase after stroke [19].

**Figure 1:**
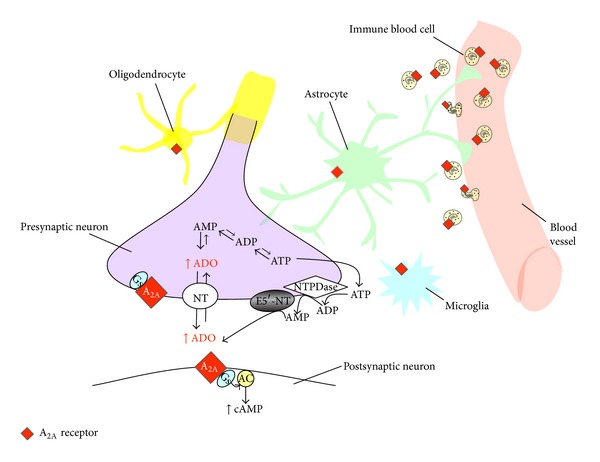
Schematic drawing of adenosine A_2A_ receptor localization on different cell types. Adenosine A_2A_ receptors are expressed at central level on presynaptic and postsynaptic neurons, on astrocytes, on microglia, and on oligodendrocytes. A_2A_ receptors are present also at peripheral level on leukocytes and vasculature. After cerebral ischemia, leukocytes infiltrate into ischemic tissue due to increased permeability of blood-brain barrier (BBB). During ischemia, extracellular adenosine level increases mainly due to (i) extracellular ATP degradation by NTPDases; (ii) release of adenosine* per se* from cells likely by the equilibrative nucleoside transporter (ENT); (iii) inhibition of adenosine-uptake processes due to downregulation of concentrative nucleoside transporters (CNT) 2 and 3 and of ENT. AC: adenylate cyclase; ADO: adenosine; ADP: adenosine diphosphate; AMP: adenosine monophosphate; ATP: adenosine triphosphate; cAMP: cyclic adenosine monophosphate; E5′-NT: ecto-5′-nucleotidase; NT: nucleoside transporter; Gs: stimulatory *G*-protein; NTDPase: ecto-nucleoside triphosphate diphosphohydrolases. The proportions of the various components of the nervous tissue have not been kept.

Numerous authors have indicated adenosine and its receptors as a target for therapeutic implementation in the treatment of stroke. Extracellular adenosine acts through multiple *G*-protein coupled receptors (adenosine receptor subtypes A_1_, A_2A_, A_2B_, and A_3_) to exert a variety of physiological effects [20]. Adenosine receptors are expressed at significant levels in neurons and glial cells and in inflammatory cells (such as lymphocytes and granulocytes) [21–26] (Figure 1). The wide distribution is consistent with the multifaceted neurochemical and molecular effects of adenosine receptor activation and suggests that the role of adenosine in ischemia is the consequence of an interplay among different receptor activation in neuronal, glial, and inflammatory cells, which changes depending on the time-related development of the pathological condition.

During ischemia, adenosine has long been known to act predominantly as a neuroprotectant endogenous agent [27–32]. Adenosine infusion into the ischemic striatum has been shown to significantly ameliorate neurological outcome and reduce infarct volume after transient focal cerebral ischemia [33]. Protective effects are greatly attributed to A_1_ receptor activation due to reduced Ca^2+^ influx, thus lowering presynaptic release of excitatory neurotransmitters [33–38] and in particular of glutamate which exerts an excitotoxic effect during ischemia mainly by overstimulation of NMDA receptors [39]. In addition, by directly increasing the K^+^ and Cl^−^ ion conductances, adenosine stabilises the neuronal membrane potentials, thus reducing neuronal excitability [39]. Consequent reduction in cellular metabolism and energy consumption [40] and moderate lowering of the body/brain temperature [41] protect against ischemia.

Although data demonstrate a neuroprotective effect of adenosine through A_1_ receptors during ischemia, the use of selective A_1_ agonists is hampered by undesirable effects such as sedation, bradycardia, and hypotension [42, 43]. More recently adenosine A_2A_ receptors emerged as an interesting target in ischemia.

We largely limit our overview to the A_2A_ adenosine receptor subtype in brain whose new insights are into control of excitotoxicity and neuroinflammation phenomena in ischemia. In this paper, we summarize recent developments that have contributed to the understanding of how this adenosine receptor subtype modulates tissue damage in brain ischemia models. A list of A_2A_ receptor ligands used in different* “in vitro”* and* “in vivo”* hypoxia/ischemia models is provided in Table 1.

## 2. Adenosine *A*_2A_ Receptor Antagonists Protect against Primary Ischemic Injury

### 2.1. *A*_2A_ Receptor Antagonists Are Protective against Ischemic Damage

Gao and Phillis [50] demonstrated for the first time that the nonselective A_2A_ receptor antagonist, 9-chloro-2-(2-furanyl)-[1,2,4] triazolo[1,5-c]quinazolin-5-amine (CGS15943), reduced cerebral ischemic injury in the gerbil following global forebrain ischemia. Thereafter many reports have confirmed the neuroprotective role of A_2A_ receptor antagonists in different models of ischemia. The selective A_2A_ receptor antagonist, 8-(3-chlorostyryl) caffeine (CSC), as well as the less selective antagonists, CGS15943 and 4-amino [1,2,4] triazolo [4,3a] quinoxalines (CP66713), both administered before ischemia, protected against hippocampal cell injury during global forebrain ischemia in gerbils [49, 52]. The selective A_2A_ receptor antagonist, 4-(2-[7-amino-2-(2-furyl) [1,2,4] triazolo[2,3-a][1,3,5] triazin-5-yl-amino]ethyl) phenol (ZM241385), administered before ischemia, reduced hippocampal injury and improved performance in the Morris water maze in hyperglycemic four-vessel occluded rats [54]. In all the mentioned studies, adenosine A_2A_ receptor antagonists were administered before ischemia. Relevantly to a possible clinical use of drugs in stroke, in subsequent studies, A_2A_ antagonists were administered after ischemia. The selective A_2A_ receptor antagonist, 7-(2-phenylethyl)-5-amino-2-(2-furyl)-pyrazolo-[4,3-e]-1,2,4,triazolo[1,5-c]pyrimidine (SCH58261), acutely administered after hypoxia/ischemia in neonatal rats [57] and soon after focal ischemia in adult rats [58, 59] reduced brain damage 24 hours thereafter. The same antagonist, administered subchronically, was protective against brain damage, neurological deficit [60, 61, 67], and disorganization of myelin [61] 24 hours after focal cerebral ischemia in the adult rat. In the model of global ischemia (i.e., 7 min asphyxic cardiac arrest) in newborn piglets, posttreatment with SCH58261, infused soon after resuscitation and for 6 hours, improved neurologic recovery and protected striatopallidal neurons after 4 days from ischemia [63]. SCH58261 behaves as a significant protective agent at a dose (0.01 mg/kg) that does not have cardiovascular effects. This low dose does not affect motor activity in naive animals but decreases contralateral turning behaviour after monolateral middle cerebral artery occlusion (MCAo) induced by the monofilament technique [59, 60]. At a higher dose, in the range that is effective in different models of Parkinson's Disease (PD), the same drug significantly increases motility and rearing in the rat [68]. A noxious role of A_2A_ receptors during ischemia is supported by the observation that A_2A_ receptor knock-out (KO) mice show significantly decreased infarct volumes after focal cerebral ischemia when compared with their wild-type littermates [69, 70].

Most recently, the question has been raised if A_2A_ receptor continuous blockade over an extended time-window after ischemia is protective. CSC continuously administered over 72 hours, using subcutaneously implanted osmotic minipumps, after permanent MCAo in spontaneously hypertensive rats, did not decrease brain infarct volume determined by magnetic resonance imaging 3 days after induction of ischemia [53]. Authors attributed the lack of protection to high hepatic metabolism and elimination of CSC [53]. Consistently, Melani and coworkers (unpublished observation) found a lack of protection on infarct volume by SCH58261 administered subchronically (three times in the first day) or chronically (twice/day for 7 days) 7 days after 1 hour transient MCAo.

### 2.2. *A*_2A_ Receptor Antagonism Protects from the Increase of Glutamate Extracellular Concentrations and NMDA Receptor Function

A_2A_ receptors are expressed on neurons at high levels in the striatum [71] and at lower levels in all other brain regions as detected by autoradiography [72] and real time PCR [73]. A_2A_ receptors in the striatum are mostly present on GABA-enkephalin neurons [74] but are also located presynaptically [25, 75, 76] on glutamatergic terminals [77] where they can directly regulate glutamate outflow under normoxic [78, 79] and ischemic conditions [65, 66]. Adenosine, by A_2A_ receptor stimulation, promotes glutamate release under normoxic and ischemic conditions* in vivo* [44, 51, 80–82]. Consistently, A_2A_ receptors play an important modulation of synaptic transmission [83, 84] as mostly demonstrated in the hippocampus [85–87]. In the CA1 area of the rat hippocampus, which is the most sensitive region to ischemia, the selective A_2A_ receptor agonist, CGS21680, clearly reduces the depression of synaptic activity brought about by OGD [47]. Following A_2A_ receptor stimulation the increase of extracellular glutamate concentration counteracts depression brought about by adenosine A_1_ receptors. In agreement, the selective A_2A_ receptor antagonists, ZM241385 and SCH58261, delay the appearance of anoxic depolarization (AD), a phenomenon strictly related to cell damage and death [88], protect from the synaptic activity depression brought about by a severe (7 min) OGD period, and protect CA1 neuron and astrocyte from injury [55]. Same effects of ZM241385 were observed after a severe 9 min OGD period in the gyrus dentatus of the hippocampus [56]. The time-window of the protective effects of the A_2A_ receptor antagonists in the hippocampus overlaps with the delay obtained by treating the slices with glutamate receptor antagonists [89, 90], indicating that their effects are attributable to reduced glutamate excitotoxicity.

Several mechanisms contribute to the A_2A_ receptor regulation of extracellular glutamate concentrations. A_2A_ receptor stimulation might regulate extracellular glutamate not only by reducing release from glutamatergic terminals but also by modulation of glutamate uptake transporter. In the brain, adenosine A_2A_ receptors are expressed on both neurons and glia [21, 71]. In particular, A_2A_ receptors located on astrocytes mediate inhibition of glutamate uptake by glutamate transporter-1 (GLT-1) [91–93]. Recent data show that while acute exposure to the selective A_2A_ receptor agonist, CGS21680, reduces glutamate uptake, prolonged exposure to the same agonist inhibits GLT-1 and glutamate-aspartate transporter mRNA and protein levels from astrocytes [94]. Such inhibition is exerted through modulation of Na^+^/K^+^-ATPase [95]. An imbalance of A_1_/A_2A_ receptor expression might also contribute to inhibition of excitatory synaptic transmission under ischemia. Short periods of global ischemia decrease A_1_ adenosine receptor density in the brain likely due to an internalization of A_1_ adenosine receptors in nerve terminals [96]. Moreover tight A_1_/A_2A_ receptor interaction exists. In hippocampal and cortical nerve terminals A_2A_ receptors might increase glutamate outflow by a protein kinase C-mediated decrease of the affinity of A_1_ receptors [97]. A heteromerization of adenosines A_1_ and A_2A_ receptors in striatal glutamatergic nerve terminals might allow adenosine to exert a fine-tuning modulation of glutamatergic neurotransmission. A main biochemical characteristic of the A_1_/A_2_ receptor heteromer is the ability of A_2A_ receptor activation to reduce the affinity of the A_1_ receptor for agonists with an ultimate switch mechanism by which low and high concentrations of adenosine inhibit and stimulate, respectively, glutamate release [98].

Adenosine acting on A_2A_ receptors is such an important modulatory substance by controlling synaptic transmission and also by regulating AMPA [99] and NMDA receptor function [100]. In striatal membranes, the NMDA-mediated excitation, leading to a depolarized plateau potential and spike firing, is regulated by dopamine and adenosine acting at D_2_ and A_2A_ receptor heteromers that regulate Ca^++^ channel activity through mechanisms relying upon specific protein-protein interactions [101]. A_2A_ receptor chronic blockade by treatment with SCH58261 induces a remodeling of NR1 and NR2A/NR2B subunit expression of NMDA receptors in the striatum of Huntington transgenic mice [102]. Moreover, given that mGlu5 receptors “set the tone” of NMDA receptor-mediated neurotransmission [103], it appears important that mGlu5 receptors are under the tight control of A_2A_ receptors [100]. In the hippocampus A_2A_ and mGlu5 receptors are colocated and A_2A_ receptors play a permissive role in mGlu5 receptor-mediated potentiation of NMDA effects [104]. Such modulations by A_2A_ receptors might be relevant in pathological conditions such as ischemia. By the use of SCH58261, it was demonstrated that A_2A_ receptors support the expression and recruitment of calcium-permeable AMPA receptors during LTP induced by OGD in rat hippocampal slices [64]. In a model of global ischemia in newborn piglets (7 min Asphyxic Cardiac Arrest), inhibition of phosphorylation of NMDA receptor NR1 subunit and inhibition of Na^+^/K^+^-ATPase and of cAMP-regulated phosphoprotein 32 kDa (DARPP32) might also account for protective effect of the selective A_2A_ receptor antagonist SCH58261 [63]. The ability of adenosine A_2A_ receptors in controlling glutamate receptor functions might represent an attractive mechanism in protecting against acute excitoxicity after ischemia. In fact, in a number of* in vitro* and* in vivo* experimental models of ischemia, glutamate receptor antagonists, acting either on NMDA receptor or on group I metabotropic receptors, are effective neuroprotective agents; none of the glutamate receptor antagonists tested in clinical trials showed positive results or had an acceptable benefit/side effects ratio [105].

*In vivo*, a definite overexpression of A_2A_ receptors was found in neurons of the striatum and cortex 24 hours after focal ischemia [106] and, in* in vivo* experiments, the low dose of SCH58261 that protects against tissue damage induced by MCAo or quinolinic acid (QA) excitotoxicity also reduces glutamate extracellular concentrations estimated by microdialysis [59, 107]. This supports that protective effects of low doses of A_2A_ receptor antagonists administered early after brain ischemia are largely due to reduced excitotoxicity and to the ensuing excitotoxic cascade attributable to stimulation of NMDA receptors [59]. The robust protection by A_2A_ receptor antagonism is consistent with the observation that adenosine A_2A_ receptor KO mice are protected from an excess of striatal glutamate outflow and damage induced by transient MCAo [69, 70].

A further protective effect of A_2A_ receptor antagonism may be attributed to the capability of increasing GABA outflow during ischemia. The major part of excitatory glutamatergic innervation is modulated by inhibitory GABA-releasing interneurons. Potentiation of GABAergic synaptic transmission has neuroprotective effects in several experimental models of cerebral ischemia [108]. GABA is strongly increased in the cortex and striatum during ischemia [15, 109] and evidence shows that selective A_2A_ receptor stimulation decreases ischemia-evoked GABA outflow [109, 110] and enhances GABA transport into nerve terminals by restraining PKC inhibition of GAT-1 [111].

The neuroprotective properties of A_2A_ receptor antagonists largely reside in effects mediated by A_2A_ receptors located on brain cells, in particular in control of excitotoxicity as demonstrated by the observation that the A_2A_ receptor selective antagonist, ZM241385, injected peripherally or directly intra-hippocampus is protective against excitotoxicity induced by kainate [48] and by the combinations of quinolinic acid and IL-1*β* [112].

### 2.3. *A*_2A_ Receptor Antagonists Protect from Ischemia–Induced Activation of Mitogen-Activated Protein Kinases (MAPKs) and c-fos Expression

Several data indicate that regulation of proteins involved in transcriptional or post-translational mechanisms plays an important role in the neuroprotective effect of A_2A_ receptor antagonism in ischemia.

All members of the MAPKs family are activated up to 24 hours after ischemia [113, 114]. p38 and ERK1/2 are activated in neurons and in microglia [60, 113, 115, 116]. A definite overexpression of A_2A_ receptors was found not only in neurons but also on microglia of the ischemic tissue 24 hours after focal ischemia [106]. Subchronic administration of the A_2A_ receptor antagonist, SCH58261, reduced phospho-p38 in microglia while it did not affect ERK1/2 activation [60]. It is known that soon after excitotoxic phenomena, resident microglial cells initiate a rapid change in their phenotype that is referred to as microglial cell activation [117] and, by producing cytotoxic substances and cytokines, start an inflammatory response that exacerbate brain damage [6]. Since inhibition of p38 activation has direct neuroprotective effects in hippocampal brain slices after OGD [118], a control of p38 activation by A_2A_ receptor antagonism [60] might account for protection after ischemia. Such results are in agreement with the result that intracerebroventricular injection of SCH58261 prevents the recruitment of activated microglial cells and the increase in IL-1*β* evaluated 4 hours after intraperitoneal administration of lipopolysaccharide (LPS) [119]. It is also important to consider that A_2A_ receptor antagonists are effective in preventing neurotoxicity in isolated glia. A_2A_ receptor stimulation is known in fact to cause activation of microglia [120] and A_2A_ receptor antagonists have been shown to suppress microglia activation in murine N9 microglial cells exposed to an inflammatory stimulus such as LPS [121]. A_2A_ receptor antagonist suppresses the CGS21680-induced potentiation of LPS-induced NO release from mixed glial cultures as well [122]. Overall results indicate that A_2A_ receptors present on microglial cells are pivotal in mediating a secondary damage consisting in neuroinflammation (see later in the paper) after ischemia.

Twenty-four hours after MCAo, subchronic administration of the A_2A_ receptor antagonist, SCH58261, also reduces phospho-JNK, that is expressed in few neurons, but mainly in mature oligodendrocytes and in oligodendrocyte precursors (OPCs) (stained by Olig2 and NG2 antibodies) [61, 123]. Phospho-JNK is a factor involved in oligodendrocyte death [124, 125]. Interestingly activation of JNK has been described in oligodendrocytes in multiple sclerosis lesions where oligodendrocytes are major targets of the disease [126]. A specific peptide inhibitor of JNK protects against cell death induced by OGD* in vitro* [127] and by MCAo* in vivo* [127, 128]. JNK2/3 KO mice are protected from damage following cerebral ischemia [129, 130]. Therefore we must assume that JNK activation in oligodendrocytes and neurons represents a noxious event after ischemia that can damage oligodendrocytes bringing to myelin damage and disorganization [61]. A_2A_ receptor antagonism also reduces Olig2 [61] that is a transcription factor expressed mostly by OPC while mature oligodendrocytes are characterized by lower levels of Olig2 [131]. Data have suggested that A_2A_ antagonism stimulates OPC differentiation to mature cells after ischemia. In agreement we have recently reported that, in primary OPC culture, selective stimulation of A_2A_ receptors by CGS21680 inhibits maturation of OPC in the firsts 10 days of* in vitro* differentiation [132]. The drug also inhibits K^+^ “delayed rectifier” channels (KDR) [132] that are known to inhibit proliferation and differentiation of OPC to mature oligodendrocytes, thus preventing myelin deposition [133, 134].

Besides a direct effect of the A_2A_ receptor antagonists on A_2A_ receptors located on oligodendrocytes or microglia, we must consider that the reduced MAPK activation by SCH58261, in the initial hours after* in vivo* ischemia, is secondary, to overall reduction in the excitotoxic cascade that in turn primes MAPK activation [59]. In fact, oligodendroglia are extremely sensitive to glutamate receptor overactivation and ensuing oxidative stress [135–137] as well as to cytokines [138] and p38 activation is definitely induced by NMDA receptor stimulation in cerebellar granule cells [139] and in spinal cord cultures [140].

It is of note that, twenty-four hours after permanent MCAo, the A_2A_ antagonist, SCH58261, also reduces gene c-fos expression in glial cells [62]. Products of the Fos family are players in inducing inflammatory gene expression in glial cells [141].

## 3. Adenosine *A*_2A_ Receptor Agonists Protect against Secondary Injury

### 3.1. *A*_2A_ Receptor Agonists Are Protective against Ischemic Damage

While many data support that A_2A_ receptor antagonists protect against central excitotoxicity, the protective effect of A_2A_ receptor agonists appears attributable to different mechanisms. The A_2A_ receptor antagonist ZM241385 administered repeatedly (1 mg/kg i.p.) in the 12 hours after traumatic brain injury was protective 15 min after trauma when cerebro spinal fluid (CSF) glutamate concentration rose; conversely, the A_2A_ receptor agonist, CGS21680, administered repeatedly (0.1 mg/kg i.p.) in the 12 hours after trauma was protective 3 hours after trauma when CSF glutamate concentrations were down [142].

A protective role of adenosine A_2A_ receptor in hypoxia/ischemia was demonstrated in newborn rodents. A_2A_ receptor KO neonatal mice show aggravated hypoxic/ischemic injury in comparison to wild-type mice [143] and, in immature brain forebrain slices, it was demonstrated that cannabinoids induce robust neuroprotection through both CB(2) and A_2A_ adenosine receptors [144]. Most recently it was demonstrated that A_2A_ receptor KO mice subjected to chronic cerebral hypoperfusion by permanent stenosis of bilateral common carotid artery show impairment in working memory, increased demyelination, proliferation of glia, and increased levels of proinflammatory cytokines [145]. In adult gerbil, a protective effect of adenosine A_2A_ receptor agonists was reported by Von Lubitz et al. [49] who demonstrated that the A_2A_ receptor agonist, APEC, administered systemically before a global 10 min ischemia, ameliorated recovery of blood flow and animal and neuron survival. Moreover Sheardown and Knutsen [45] demonstrated that a high dose of the selective A_2A_ receptor agonist, CGS21680 (10 mg/kg i.p.), administered after 5 min of global ischemia in gerbil, exhibited highly significant protection against neuronal loss, but was inactive at 3 mg/kg. In these two works in adult gerbils, adenosine agonists were administered before ischemia or at a high dose. In considering translation to clinic, a main problem of A_2A_ receptor agonists is their cardiovascular effect: adenosine A_2A_ receptors located on vase smooth muscle and endotelial cells exert a vasodilatory effect [146]. Consistently A_2A_ receptor agonists might induce hypotension and increase hearth rate. Schindler and coworkers [147, 148] reported that the decrease of blood pressure induced by 0.5 mg/kg i.p. CGS21680 in conscious rats is most probably mediated in the periphery, while the increase of heart rate is mediated at central level. We recently demonstrated that the selective A_2A_ receptor agonist, CGS21680, at dose of 0.1 mg/kg i.p., increased heart rate only in the first hour after administration, but no effect on blood pressure or on heart rate was observed at the lower dose of 0.01 mg/kg [46]. Relevantly our recent experiments have demonstrated that the A_2A_ receptor agonist, CGS21680, administered twice/day for 7 days (chronic protocol) at dose of 0.01 and 0.1 mg/kg, starting 4 hours after transient (1 hour) MCAo, induced protection from neurological deficit, weight loss, cortical infarct volume, myelin disorganization and glial activation [46]. Protective effect is exerted only when CGS21680 is chronically administered. In fact the A_2A_ receptor agonist administered at the same dose (0.1 mg/kg) but in a shorter therapeutic window (4 and 20 hours after induction of MCAo, subchronic protocol) has not reduced the infarct volume 24 hours after permanent MCAo nor 7 days after transient MCAo (unpublished data; see Table 2). The protective effects of chronic administration of CGS21680 at dose of 0.01 and 0.1 mg/kg neither can be attributed to changes in the cardiovascular parameters either at peripheral or central level nor can be attributed to direct effects on motility because CGS21680 at these low doses does not affect motor behavior of rats [149].

Several mechanisms might account for protection by A_2A_ receptor stimulation by direct effects on brain cells. In a rat model of intracerebral hemorrhage, CGS21680 administered directly into the striatum immediately prior to the induction of intracerebral hemorrhage reduces parenchymal neutrophile infiltration and tissue damage: an effect that might be mediated by inhibition of TNF-*α* expression [150]. Moreover, activation of central A_2A_ receptors is known to increase expression and release of neurotrophic factors [151] as NGF in microglia [152], BDNF in mice hippocampus [153], in rat cortical neurons [154], and in primary cultures of microglia [121], and GDNF in striatal neurons [155]. Consistently it was recently demonstrated that* in vivo* chronic oral administration of the A_2A_ receptor antagonist, KW-6002, decreases both mRNA and protein levels of BDNF receptor (TrkB-FL) and its signaling in the hippocampal CA1 area [156]. The increase in neurotrophic factor expression by adenosine A_2A_ receptor stimulation may contribute to restore neurological functions and cerebral damage after brain ischemia. We must also remember that adenosine is implicated in cerebral blood flow regulation as a vasodilator agent acting on A_2A_ receptors on endothelial cells of brain vessels, thus favouring brain perfusion [146].

Several lines of evidence in excitotoxicity and spinal cord trauma* in vivo* models do not support, however, that protection by A_2A_ receptor agonists is exerted at A_2A_ receptors located on CNS cells. Jones and coworkers [157] showed that peripheral administration of the A_2A_ receptor agonist, CGS21680, protected the hippocampus against kainate-induced excitotoxicity while the direct injection of CGS21680 into the hippocampus failed to afford protection [157]. Similar results were obtained after spinal cord trauma where CGS21680 protected from damage when injected systemically but not when centrally injected into the injured spinal cord [158].

### 3.2. *A*_2A_ Receptor and Neuroinflammation

Minutes to hours after onset of cerebral ischemia, a cascade of inflammatory events is initiated through activation of resident cells [159]. The early massive increase in extracellular glutamate after ischemia has a main role in activating resident immune cells and producing mediators of inflammation [6]. Immunity and inflammation are key elements of the pathology of stroke. Recent developments have revealed that stroke engaged both innate and adaptative immunity. Molecules generated by cerebral ischemic tissue activate components of innate immunity, promote inflammatory signaling, and contribute to tissue damage. The A_2A_ adenosine receptors are expressed both on cells of innate (microglia, macrophages, mast cells, monocytes, dendritic cells, and neutrophils) and on cells of adaptive (lymphocytes) immunity [160, 161]. Soon after excitotoxic phenomena, microglial cells initiate a rapid change in their phenotype [60, 119] that is referred to as microglial cell activation [117]. Microglia typically respond with proliferation, migration, and production of inflammatory substances to viral or bacterial stimuli or to cell damage and degeneration [121, 162] and, by producing cytotoxic substances, cytokines (TNF-*α*, IL-1*β*) [119, 120, 163], and chemokines, contribute to the inflammatory response that follows ischemic insult, further exacerbating brain damage [6]. Proinflammatory mediators and oxidative stress contribute to the endothelial expression of cellular adhesion molecules [7, 8] and to an altered permeability of the blood-brain barrier (BBB) that promotes the infiltration of leukocytes (neutrophils, lymphocytes, and monocytes) [164] in the brain ischemic tissue.

In a model of transient focal cerebral ischemia induced by MCAo, definite microglial activation is present after 12 hours [165]. After ischemia, although reperfusion is necessary for tissue survival, it also contributes to additional tissue damage. Under reperfusion, there is an initial increase of BBB permeability (see [166]) followed by a biphasic increase at 5 and 72 hours [167]. Changes in BBB permeability are responsible for cell infiltration. The nature of BBB permeability is dependent on the duration of ischemia, the degree of reperfusion, and the animal stroke model. Studies in the human brain after ischemic stroke confirm that neutrophils intensively accumulate in the regions of cerebral infarction [6, 168]. Selective immunostaining for granulocytes, by anti-HIS-48 antibody, shows numerous infiltrated cells in ischemic striatal and cortical core two days after tMCAo, while seven days thereafter infiltrated blood cells were not anymore observed [46]. Three days after tMCAo the majority of immune cells are neutrophils and at less extent lymphocytes [165, 169]. After tMCAo, a peak of neutrophil infiltration occurs at 6 and 48 hours thereafter [169]. Infiltrated neutrophils expressing cytokines and chemotactic factors promote expansion of the inflammatory response in ischemic tissue [160]. Correlations among neutrophil accumulation, severity of brain tissue damage, and neurological outcome have been reported by Akopov et al. [168]. Neuroinflammation is now recognized as a predominant mechanism of secondary progression of brain injury after ischemia.

Two days after MCAo, chronic treatment with the A_2A_ adenosine receptor agonist, CGS21680, has definitely reduced the number of infiltrated blood cells in the ischemic areas [46]. These results are in agreement with previous observations that A_2A_ receptor agonists systemically administered after spinal cord injury in mice protect from neurological and tissue damage, reduce inflammation parameters and blood cell infiltration [170–172]. An unequivocal role of A_2A_ receptor in controlling blood cell infiltration was demonstrated also in a model of autoimmune encephalomyelitis: A_2A_ receptor KO mice displayed increased inflammatory cell infiltration, higher neurological deficit scores and increase of different neuroinflammation parameters [173].

A bulk of evidence indicate that bone marrow-derived cells (BMDCs) are targets of A_2A_ receptor agonist protective effects. Li et al. [174] demonstrated that the protective effect against motor deficits of A_2A_ receptor agonists, systemically administered after spinal trauma, is lost in mice lacking A_2A_ receptors on BMDCs, but is restored in A_2A_ receptor KO mice reconstituted with A_2A_ receptors on BMDCs. Many studies have reported that selective activation of A_2A_ receptors directly on blood cells, including platelets, monocytes, some mast cells, neutrophils, and T cells, inhibits proinflammatory responses [175–177], reduces production of adhesion cell factors, and reduces neutrophil activation, thereby exerting antioxidant and anti-inflammatory effects [178]. A_2A_ receptor activation is known to reduce ischemia-induced rolling, adhesion, and transmigration of various peripheral inflammatory cells (such as lymphocytes, neutrophils) [160]. Overall results suggest that protection due to A_2A_ receptor agonists systemically and repeatedly administered after brain ischemia is strongly exerted at peripheral BMDCs resulting ultimately in reduced leukocyte infiltration and reduced inflammatory cascade at the central level. Consistent with its anti-inflammatory and immunosuppressive role, the protective effect of adenosine A_2A_ receptor stimulation has been observed in different pathologies where inflammatory process has an important role in tissue damage [124, 172, 179, 180] such as ischemia/reperfusion liver injury [181], spinal cord trauma [158], rheumatoid arthritis (RA) [182], acute lung inflammation [183], intestine ischemia/reperfusion injury [184], and experimental autoimmune encephalomyelitis [185].

By controlling brain neuroinflammation and BDNF signalling [186, 187], A_2A_ receptors might also have a potential for synaptic plasticity and neurogenetic processes after ischemia. Neuroinflammation in fact is known to result in inhibition of adult neurogenesis [188].

The notion that A_2A_ receptors on BMDCs are the target of the protective effects of A_2A_ receptor agonists should be reconciled with the information that selective inactivation of A_2A_ receptors on BMDCs (wild-type mice transplanted with A_2A_ receptor KO bone marrow cells) attenuates ischemic brain injury, inhibits inflammatory cytokines production, and increases the expression of anti-inflammatory cytokines in the ischemic brain 22 hours after 2 hours of focal ischemia induced by MCAo. This neuroprotection however cannot be explained by altered infiltration of the major inflammatory cells, neutrophils and microglial cells, in the ischemic brain and remains to be clarified [189].

## 4. Caffeine Consumption and Stroke Incidence 

It has been reported that acute coffee consumption is associated with increased risk of ischemic stroke in the subsequent hour in infrequent coffee drinkers (<1 cup) [190]. The increased risk might be related within hours after consumption to acute deleterious effects of the unselective A_1_/A_2A_ receptor antagonist, caffeine, that increases circulating norepinephrine [191], rises mean blood pressure [192], increases arterial stiffness [193], and impairs endothelium-dependent vasodilation [194].

More studies have instead investigated the effect of habitual consumption of caffeine on the risk of stroke. Controversial results, mainly in relation to the dose intake, were obtained [195]. A study showed that the long-term moderate consumption of coffee can provide protective effects (reducing the risk of both coronary heart disease and stroke by 10%–20%) in healthy individuals yet detrimental effects when intake was high [196]. In agreement, Larsson and Orsini [197] reported that it is the moderate coffee consumption (3-4 cups/day) that reduces the risk of stroke. Additionally, one study showed that coffee consumption (more than 4 cups/day) in men was not associated with increased risk of stroke [198] while studies performed in Swedish and USA women have indicated that habitual intake of coffee (from 1 to 5 or more cups/day) was associated with a statistically significant lower risk of total stroke [199], cerebral infarction, and subarachnoid hemorrhage but not intracerebral hemorrhage [200]. In contrast, an epidemiological study showed that neither the high (more than 4 cups/day) nor the low doses (less than 2 cups/day) have the most dangerous effect but it is the intermediate consumption (2–4 cups/day) of coffee which can be the most harmful [201]. Thus the effect of different consumption of caffeine in reducing the risk of ischemic stroke still demand further study.

## 5. *A*_2A_ Receptor Based Therapies in Cerebral Ischemia

Evidence reported up to now indicate that antagonism or stimulation of A_2A_ receptors might be a protective strategy secondary to the time-related development of phenomena typical of ischemia. After ischemia, extracellular glutamate concentrations remain elevated at least up to 4 hours after permanent MCAo [15, 59] and up to 12 hours after brain trauma [142]. The massive increase of glutamate excitotoxicity triggers acute tissue injury and the start of an inflammatory cascade that is stressed by blood cell infiltration. While central A_2A_ receptors in the first hours after ischemia are critical in increasing glutamate extracellular concentrations, A_2A_ receptors on blood cells are critical hours and days after ischemia in decreasing activation, adhesion, and infiltration of blood cells in brain parenchyma. Altogether, evidence suggests that A_2A_ receptor antagonists provide protection centrally by reducing excitotoxicity, while A_2A_ receptor agonists provide protection by acting on blood cells controlling massive infiltration and neuroinflammation in the hours after brain ischemia. In agreement the lack of detecting a protection by A_2A_ receptor antagonism at later time after stroke [53, our unpublished observation] might be attributable to the fact that protection is overwhelmed by subsequent damage brought about by blood cell infiltration that starts 6 hours after ischemia and peaks at 2 days thereafter [46, 165, 169].

These observations highlight that a therapeutic strategy with adenosine A_2A_ receptor antagonists/agonists should be carefully evaluated in terms of time after ischemia. When considering use of adenosine A_2A_ receptor active drugs to protect against brain ischemia, attention should be given to administration time after injury and to the dose used. In fact A_2A_ receptors located on endothelial cells mediate important effect on systemic blood pressure and heart frequency. However both A_2A_ receptor antagonists [58, 59, 61] and agonists [46] are protective in ischemia models at doses that do not modify blood pressure nor the heart frequency.

The design and development of new adenosine A_2A_ receptor ligands is an area of intense research activity [202, 203].

## 6. Conclusions 

Under neurodegenerative conditions involving ischemia, excitotoxicity is a first phenomenon. Thereafter, the interplay of resident glial cells with infiltrating peripheral BMDCs produces neuroinflammation. On the light that the role of adenosine A_2A_ receptors in ischemia is not univocal, it is important to clarify the windows in which A_2A_ receptors play a noxious or protective role after ischemia. This will be important to devise a correct therapeutic strategy with antagonists and/or agonists at this receptor. Considering translation to clinical practice, a very short time-window of minutes/few hours would be available for A_2A_ receptor antagonists after stroke, while a focus on inflammatory responses to stroke provides a wide therapeutic time-window of hours and even days after stroke for adenosine A_2A_ receptor agonists. A novel therapeutic strategy could involve, when possible, early treatment with A_2A_ receptor antagonists to reduce excitotoxicity followed by adenosine A_2A_ receptor agonist treatment for the control of later secondary injury.

## Figures and Tables

**Table 1:** Adenosine A_2A_ receptor ligands used in brain ischemia *“in vivo”* and *“in vitro”* models.

	Brain ischemia model	References
Adenosine A_2A_ receptor agonists		
CGS21680	Global ischemia in rat	[44]
	Global ischemia in gerbil	[45]
	Focal ischemia in rat	[46]
	OGD hippocampal slices	[47, 48]
APEC	Global ischemia in gerbil	[49]
Adenosine A_2A_ receptor antagonists		
CGS15943	Global ischemia in gerbil	[50]
	Global ischemia in rat	[51]
CSC	Global ischemia in gerbil	[49, 52]
	Focal ischemia in hypertensive rat	[53]
CP66713	Global ischemia in gerbil	[52]
ZM241385	Global ischemia in rat	[54]
	OGD hippocampal slices	[55, 56]
SCH58261	Hypoxia/ischemia in neonatal rat	[57]
	Focal ischemia in rat	[58–62]
	Global ischemia in newborn piglet	[63]
	OGD hippocampal slices	[55, 64]
	OGD cerebrocortical slices	[65, 66]
DMPX	OGD hippocampal slices	[48]

APEC: 2-[(2-aminoethylamino)-carbonylethylphenylethylamino]-5′-N-ethylcarboxoamidoadenosine; CGS15943: 9-chloro-2-(2-furanyl)-[1,2,4]triazolo[1,5-c]quinazolin-5-amine; CGS21680: 2-p-(2-Carboxyethyl)phenethylamino-5′-N-ethylcarboxamidoadenosine; CP66713: 4-amino[1,2,4]triazolo[4,3a]quinoxalines; CSC: 8-(3-chlorostyryl)caffeine; DMPX: 3,7-dimethyl-1-propargylxanthine; OGD: oxygen and glucose deprivation; SCH58261: 7-(2-phenylethyl)-5-amino-2-(2-furyl)-pyrazolo-[4,3-e]-[1,2,4]triazolo[1,5-c]pyrimidine; ZM241385: 4-(2-[7-amino-2-(2-furyl)[1,2,4]triazolo[2,3-a][1,3,5]triazin-5-yl-amino]ethyl)phenol.

**Table 2:** Effect of A_2A_ receptor agonist, CGS21680, in acute and delayed phase of stroke.

Drug	24 h after pMCAo		7 days after tMCAo		
	Infarct volume (mm^3^)		Treatment	Infarct volume (mm^3^)	
	Cortex	Striatum		Cortex	Striatum
Vehicle	69.43 ± 1.87	46.03 ± 2.7	Subchronic	75.1 ± 5.1	28.4 ± 2.2
			Chronic	76.2 ± 4.3	31.3 ± 2.6

CGS21680	61.37 ± 8.26	45.68 ± 2.6	Subchronic 0.1	62.7 ± 5.8	30.5 ± 2.5
			Chronic 0.1	48.6 ± 9.5^#^	27.3 ± 1.7
			Chronic 0.01	51.9 ± 10.4^#^	20.9 ± 3.8

Data are the mean ± S.E.M. of *n* = 6–8 animals. In the model of permanent MCAo (pMCAo), CGS21680 was administered at the dose of 0.1 mg/kg (i.p.) after 4 h and 20 h from ischemia induction. The infarct volume was evaluated 24 h thereafter.

In the model of transient MCAo (tMCAo), CGS21680 was administered *in subchronic protocol* 4 h and 20 h after ischemia at the dose of 0.1 mg/kg (i.p.) and *in chronic protocol* starting 4 h after ischemia, at the dose of 0.01 or 0.1 mg/kg (i.p.), twice/day for 7 days. The infarct volume was evaluated 7 days after MCAo.

One-way ANOVA: ^#^ *P* < 0.05 versus chronic vehicle-treated rats.

Effects of CGS21680 chronically administered are published [46].

## References

[1] Soler EP, Ruiz VC (2010). Epidemiology and risk factors of cerebral ischemia and ischemic heart diseases: similarities and differences. *Current Cardiology Reviews*.

[2] Chen F, Qi Z, Luo Y (2014). Non-pharmaceutical therapies for stroke: mechanisms and clinical implications. *Progress in Neurobiology*.

[3] de Keyser J, Sulter G, Luiten PG (1999). Clinical trials with neuroprotective drugs in acute ischaemic stroke: are we doing the right thing?. *Trends in Neurosciences*.

[4] O'Bryant Z, Vann KT, Xiong ZG (2014). Translational strategies for neuroprotection in ischemic stroke—focusing on acid-sensing ion channel 1a. *Translational Stroke Research*.

[5] Lai TW, Zhang S, Wang YT (2014). Excitotoxicity and stroke: identifying novel targets for neuroprotection. *Progress in Neurobiology C*.

[6] Dirnagl U, Iadecola C, Moskowitz MA (1999). Pathobiology of ischaemic stroke: an integrated view. *Trends in Neurosciences*.

[7] Huang J, Upadhyay UM, Tamargo RJ (2006). Inflammation in stroke and focal cerebral ischemia. *Surgical Neurology*.

[8] Stoll G, Jander S, Schroeter M (1998). Inflammation and glial responses in ischemic brain lesions. *Progress in Neurobiology*.

[9] Phillis JW, Smith-Barbour M, O'Regan MH, Perkins LM (1994). Amino acid and purine release in rat brain following temporary middle cerebral artery occlusion. *Neurochemical Research*.

[10] Phillis JW, Smith-Barbour M, O’Regan MH (1996). Changes in extracellular amino acid neurotransmitters and purines during and following ischemias of different durations in the rat cerebral cortex. *Neurochemistry International*.

[11] Hagberg H, Andersson P, Lacarewicz J, Jacobson I, Butcher S, Sandberg M (1987). Extracellular adenosine, inosine, hypoxanthine, and xanthine in relation to tissue nucleotides and purines in rat striatum during transient ischemia. *Journal of Neurochemistry*.

[12] Dux E, Fastbom J, Ungerstedt U, Rudolphi K, Fredholm BB (1990). Protective effect of adenosine and a novel xanthine derivative propentofylline on the cell damage after bilateral carotid occlusion in the gerbil hippocampus. *Brain Research*.

[13] Matsumoto K, Graf R, Rosner G, Shimada N, Heiss W-D (1992). Flow thresholds for extracellular purine catabolite elevation in cat focal ischemia. *Brain Research*.

[14] Sciotti VM, Roche FM, Grabb MC, van Wylen DGL (1992). Adenosine receptor blockade augments interstitial fluid levels of excitatory amino acids during cerebral ischemia. *Journal of Cerebral Blood Flow and Metabolism*.

[15] Melani A, Pantoni L, Corsi C (1999). Striatal outflow of adenosine, excitatory amino acids, *γ*-aminobutyric acid, and taurine in awake freely moving rats after middle cerebral artery occlusion: correlations with neurological deficit and histopathological damage. *Stroke*.

[16] Melani A, Corti F, Stephan H (2012). Ecto-ATPase inhibition: ATP and adenosine release under physiological and ischemic in vivo conditions in the rat striatum. *Experimental Neurology*.

[17] Zimmermann H (2000). Extracellular metabolism of ATP and other nucleotides. *Naunyn-Schmiedeberg’s Archives of Pharmacology*.

[18] Fausther M, Lecka J, Soliman E (2012). Coexpression of ecto-5'-nucleotidase/CD73 with specific NTPDases differentially regulates adenosine formation in the rat liver. *The American Journal of Physiology—Gastrointestinal and Liver Physiology*.

[19] Medina-Pulido L, Molina-Arcas M, Justicia C (2013). Hypoxia and P1 receptor activation regulate the high-affinity concentrative adenosine transporter CNT2 in differentiated neuronal PC12 cells. *Biochemical Journal*.

[20] Fredholm BB, Jacobson KA, Klotz KN, Linden J (2001). International Union of Pharmacology. XXV. Nomenclature and classification of adenosine receptors. *Pharmacological Reviews*.

[21] Fiebich BL, Biber K, Lieb K (1996). Cyclooxygenase-2 expression in rat microglia is induced by adenosine A_2a_-receptors. *GLIA*.

[22] Peterfreund RA, MacCollin M, Gusella J, Stephen Fink J (1996). Characterization and expression of the human A_2a_ adenosine receptor gene. *Journal of Neurochemistry*.

[23] Brodie C, Blumberg PM, Jacobson KA (1998). Activation of the A_2A_ adenosine receptor inhibits nitric oxide production in glial cells. *FEBS Letters*.

[24] Svenningsson P, Le Moine C, Fisone G, Fredholm BB (1999). Distribution, biochemistry and function of striatal adenosine A_2A_ receptors. *Progress in Neurobiology*.

[25] Hettinger BD, Lee A, Linden J, Rosin DL (2001). Ultrastructural localization of adenosine A_2A_ receptors suggests multiple cellular sites for modulation of GABAergic neurons in rat striatum. *Journal of Comparative Neurology*.

[26] Yu L, Frith MC, Suzuki Y (2004). Characterization of genomic organization of the adenosine A_2A_ receptor gene by molecular and bioinformatics analyses. *Brain Research*.

[27] Ongini E, Adami M, Ferri C, Bertorelli R (1997). Adenosine A_2A_ receptors and neuroprotection. *Annals of the New York Academy of Sciences*.

[28] Cunha RA (2001). Adenosine as a neuromodulator and as a homeostatic regulator in the nervous system: different roles, different sources and different receptors. *Neurochemistry International*.

[29] Ribeiro JA, Sebastião AM, De Mendonça A (2002). Adenosine receptors in the nervous system: pathophysiological implications. *Progress in Neurobiology*.

[30] Schwarzschild MA, Chen J, Ascherio A (2002). Caffeinated clues and the promise of adenosine A_2a_ antagonists in PD. *Neurology*.

[31] Fredholm BB, Cunha RA, Svenningsson P (2003). Pharmacology of adenosine A_2A_ receptors and therapeutic applications. *Current Topics in Medicinal Chemistry*.

[32] Pedata F, Pugliese AM, Coppi E (2007). Adenosine in the central nervous system: effects on neurotransmission and neuroprotection. *Immunology, Endocrine and Metabolic Agents in Medicinal Chemistry*.

[33] Kitagawa H, Mori A, Shimada J, Mitsumoto Y, Kikuchi T (2002). Intracerebral adenosine infusion improves neurological outcome after transient focal ischemia in rats. *Neurological Research*.

[34] Corradetti R, Lo Conte G, Moroni F, Beatrice Passani M, Pepeu G (1984). Adenosine decreases aspartate and glutamate release from rat hippocampal slices. *European Journal of Pharmacology*.

[35] Dunwiddie TV (1984). Interactions between the effects of adenosine and calcium on synaptic responses in rat hippocampus in vitro. *Journal of Physiology*.

[36] Zetterstrom T, Fillenz M (1990). Adenosine agonists can both inhibit and enhance in vivo striatal dopamine release. *European Journal of Pharmacology*.

[37] Pedata F, Latini S, Pugliese AM, Pepeu G (1993). Investigations into the adenosine outflow from hippocampal slices evoked by ischemia-like conditions. *Journal of Neurochemistry*.

[38] Andine P (1993). Involvement of adenosine in ischemic and postischemic calcium regulation. *Molecular and Chemical Neuropathology*.

[39] Choi DW (1990). Possible mechanisms limiting N-methyl-D-aspartate receptor overactivation and the therapeutic efficacy of N-methyl-D-aspartate antagonists. *Stroke*.

[40] Greene RW, Haas HL (1991). The electrophysiology of adenosine in the mammalian central nervous system. *Progress in Neurobiology*.

[41] Gourine AV, Dale N, Gourine VN, Spyer KM (2004). Fever in systemic inflammation: roles of purines. *Frontiers in Bioscience*.

[42] White PJ, Rose'Meyer RB, Hope W (1996). Functional characterization of adenosine receptors in the nucleus tractus solitarius mediating hypotensive responses in the rat. *The British Journal of Pharmacology*.

[43] Fredholm BB, Chen J, Masino SA, Vaugeois J (2005). Actions of adenosine at its receptors in the CNS: insights from knockouts and drugs. *Annual Review of Pharmacology and Toxicology*.

[44] O'Regan MH, Simpson RE, Perkins LM, Phillis JW (1992). The selective A_2A_ adenosine receptor agonist CGS21680 enhances excitatory transmitter amino acid release from the ischemic rat cerebral cortex. *Neuroscience Letters*.

[45] Sheardown MJ, Knutsen LJS (1996). Unexpected neuroprotection observed with the adenosine A_2A_ receptor agonist CGS21680. *Drug Development Research*.

[46] Melani A, Corti F, Cellai L, Vannucchi MG, Pedata F (2014). Low doses of the selective adenosine A_2A_ receptor agonist CGS21680 are protective in a rat model of transient cerebral ischemia. *Brain Research*.

[47] Latini S, Bordoni F, Corradetti R, Pepeu G, Pedata F (1999). Effect of A_2A_ adenosine receptor stimulation and antagonism on synaptic depression induced by in vitro ischaemia in rat hippocampal slices. *British Journal of Pharmacology*.

[48] Jones PA, Smith RA, Stone TW (1998). Protection against kainate-induced excitotoxicity by adenosine A_2A_ receptor agonists and antagonists. *Neuroscience*.

[49] Von Lubitz DKJE, Lin RCS, Jacobson KA (1995). Cerebral ischemia in gerbils: effects of acute and chronic treatment with adenosine A_2A_ receptor agonist and antagonist. *European Journal of Pharmacology*.

[50] Gao Y, Phillis JW (1994). CGS 15943, An adenosine A2 receptor antagonist, reduces cerebral ischemic injury in the mongolian gerbil. *Life Sciences*.

[51] Simpson RE, O'Regan MH, Perkins LM, Phillis JW (1992). Excitatory transmitter amino acid release from the ischemic rat cerebral cortex: effects of adenosine receptor agonists and antagonists. *Journal of Neurochemistry*.

[52] Phillis JW (1995). The effects of selective A_1_ and A_2a_ adenosine receptor antagonists on cerebral ischemic injury in the gerbil. *Brain Research*.

[53] Fronz U, Deten A, Baumann F (2014). Continuous adenosine A_2A_ receptor antagonism after focal cerebral ischemia in spontaneously hypertensive rats. *Naunyn-Schmiedeberg’s Archives of Pharmacology*.

[54] Higashi H, Meno JR, Marwaha AS, Winn HR (2002). Hippocampal injury and neurobehavioral deficits following hyperglycemic cerebral ischemia: effect of theophylline and ZM 241385. *Journal of Neurosurgery*.

[55] Pugliese AM, Traini C, Cipriani S (2009). The adenosine A_2A_ receptor antagonist ZM241385 enhances neuronal survival after oxygen-glucose deprivation in rat CA1 hippocampal slices. *British Journal of Pharmacology*.

[56] Maraula G, Traini C, Mello T (2013). Effects of oxygen and glucose deprivation on synaptic transmission in rat dentate gyrus: role of A_2A_ adenosine receptors. *Neuropharmacology*.

[57] Bona E, Ådén U, Gilland E, Fredholm BB, Hagberg H (1997). Neonatal cerebral hypoxia-ischemia: the effect of adenosine receptor antagonists. *Neuropharmacology*.

[58] Monopoli A, Lozza G, Forlani A, Mattavelli A, Ongini E (1998). Blockade of adenosine A_2A_ receptors by SCH 58261 results in neuroprotective effects in cerebral ischaemia in rats. *NeuroReport*.

[59] Melani A, Pantoni L, Bordoni F (2003). The selective A_2A_ receptor antagonist SCH 58261 reduces striatal transmitter outflow, turning behavior and ischemic brain damage induced by permanent focal ischemia in the rat. *Brain Research*.

[60] Melani A, Gianfriddo M, Vannucchi MG (2006). The selective A_2A_ receptor antagonist SCH 58261 protects from neurological deficit, brain damage and activation of p38 MAPK in rat focal cerebral ischemia. *Brain Research*.

[61] Melani A, Cipriani S, Vannucchi MG (2009). Selective adenosine A_2A_ receptor antagonism reduces JNK activation in oligodendrocytes after cerebral ischaemia. *Brain*.

[62] Petroni A, Papini N, Blasevich M, Galli C (2002). Blockade of A_2A_ adenosine receptors leads to *c-fos* inhibition in a rat model of brain ischemia. *Pharmacological Research*.

[63] Yang ZJ, Wang B, Kwansa H (2013). Adenosine A_2A_ receptor contributes to ischemic brain damage in newborn piglet. *Journal of Cerebral Blood Flow and Metabolism*.

[64] Dias RB, Rombo DM, Ribeiro JA, Sebastião AM (2013). Ischemia-induced synaptic plasticity drives sustained expression of calcium-permeable AMPA receptors in the hippocampus. *Neuropharmacology*.

[65] Marcoli M, Raiteri L, Bonfanti A (2003). Sensitivity to selective adenosine A_1_ and A_2A_ receptor antagonists of the release of glutamate induced by ischemia in rat cerebrocortical slices. *Neuropharmacology*.

[66] Marcoli M, Bonfanti A, Roccatagliata P (2004). Glutamate efflux from human cerebrocortical slices during ischemia: vesicular-like mode of glutamate release and sensitivity to A_2A_ adenosine receptor blockade. *Neuropharmacology*.

[67] Pedata F, Gianfriddo M, Turchi D, Melani A (2005). The protective effect of adenosine A_2A_ receptor antagonism in cerebral ischemia. *Neurological Research*.

[68] Svenningsson P, Nomikos GG, Ongini E, Fredholm BB (1997). Antagonism of adenosine A_2A_ receptors underlies the behavioural activating effect of caffeine and is associated with reduced expression of messenger RNA for NGFI-A and NGFI-B in caudate-putamen and nucleus accumbens. *Neuroscience*.

[69] Chen JF, Huang Z, Ma J (1999). A_2A_ adenosine receptor deficiency attenuates brain injury induced by transient focal ischemia in mice. *Journal of Neuroscience*.

[70] Gui L, Duan W, Tian H (2009). Adenosine A_2A_ receptor deficiency reduces striatal glutamate outflow and attenuates brain injury induced by transient focal cerebral ischemia in mice. *Brain Research*.

[71] Fink JS, Weaver DR, Rivkees SA (1992). Molecular cloning of the rat A_2_ adenosine receptor: selective co-expression with D_2_ dopamine receptors in rat striatum. *Molecular Brain Research*.

[72] Johansson B, Fredholm BB (1995). Further characterization of the binding of the adenosine receptor agonist [^3^H]CGS 21680 to rat brain using autoradiography. *Neuropharmacology*.

[73] Dixon AK, Gubitz AK, Sirinathsinghji DJS, Richardson PJ, Freeman TC (1996). Tissue distribution of adenosine receptor mRNAs in the rat. *British Journal of Pharmacology*.

[74] Schiffmann SN, Jacobs O, Vanderhaeghen JJ (1991). Striatal restricted adenosine A2 receptor (RDC8) is expressed by enkephalin but not by substance P neurons: an in situ hybridization histochemistry study. *Journal of Neurochemistry*.

[75] Rosin DL, Hettinger BD, Lee A, Linden J (2003). Anatomy of adenosine A_2A_ receptors in brain: morphological substrates for integration of striatal function. *Neurology*.

[76] Rebola N, Canas PM, Oliveira CR, Cunha RA (2005). Different synaptic and subsynaptic localization of adenosine A_2A_ receptors in the hippocampus and striatum of the rat. *Neuroscience*.

[77] Rebola N, Rodrigues RJ, Lopes LV, Richardson PJ, Oliveira CR, Cunha RA (2005). Adenosine A_1_ and A_2A_ receptors are co-expressed in pyramidal neurons and co-localized in glutamatergic nerve terminals of the rat hippocampus. *Neuroscience*.

[78] Lopes LV, Cunha RA, Kull B, Fredholm BB, Ribeiro JA (2002). Adenosine A_2A_ receptor facilitation of hippocampal synaptic transmission is dependent on tonic A_1_ receptor inhibition. *Neuroscience*.

[79] Rodrigues RJ, Alfaro TM, Rebola N, Oliveira CR, Cunha RA (2005). Co-localization and functional interaction between adenosine A_2A_ and metabotropic group 5 receptors in glutamatergic nerve terminals of the rat striatum. *Journal of Neurochemistry*.

[80] Popoli P, Betto P, Reggio R, Ricciarello G (1995). Adenosine A_2A_ receptor stimulation enhances striatal extracellular glutamate levels in rats. *European Journal of Pharmacology*.

[81] Corsi C, Melani A, Bianchi L, Pepeu G, Pedata F (1999). Striatal A_2A_ adenosine receptors differentially regulate spontaneous and K+-evoked glutamate release in vivo in young and aged rats. *NeuroReport*.

[82] Corsi C, Melani A, Bianchi L, Pedata F (2000). Striatal A_2A_ adenosine receptor antagonism differentially modifies striatal glutamate outflow in vivo in young and aged rats. *NeuroReport*.

[83] Sebastião AM, Ribeiro JA (1996). Adenosine A_2_ receptor-mediated excitatory actions on the nervous system. *Progress in Neurobiology*.

[84] Lopes LV, Sebastião AM, Ribeiro JA (2011). Adenosine and related drugs in brain diseases: present and future in clinical trials. *Current Topics in Medicinal Chemistry*.

[85] Cunha RA, Johansson B, van der Ploeg I, Sebastião AM, Ribeiro JA, Fredholm BB (1994). Evidence for functionally important adenosine A_2A_ receptors in the rat hippocampus. *Brain Research*.

[86] Martin O'Kane E, Stone TW (1998). Interaction between adenosine A_1_ and A_2_ receptor-mediated responses in the rat hippocampus in vitro. *European Journal of Pharmacology*.

[87] Lopes LV, Cunha RA, Ribeiro JA (1999). ZM 241385, an adenosine A_2A_ receptor antagonist, inhibits hippocampal A_1_ receptor responses. *European Journal of Pharmacology*.

[88] Somjen GG (2001). Mechanisms of spreading depression and hypoxic spreading depression-like depolarization. *Physiological Reviews*.

[89] Tanaka E, Yamamoto S, Kudo Y, Mihara S, Higashi H (1997). Mechanisms underlying the rapid depolarization produced by deprivation of oxygen and glucose in rat hippocampal CA1 neurons in vitro. *Journal of Neurophysiology*.

[90] Yamamoto S, Tanaka E, Shoji Y, Kudo Y, Inokuchi H, Higashi H (1997). Factors that reverse the persistent depolarization produced by deprivation of oxygen and glucose in rat hippocampal CA1 neurons in vitro. *Journal of Neurophysiology*.

[91] Nishizaki T, Nagai K, Nomura T (2002). A new neuromodulatory pathway with a glial contribution mediated via A_2A_ adenosine receptors. *GLIA*.

[92] Pintor A, Galluzzo M, Grieco R, Pèzzola A, Reggio R, Popoli P (2004). Adenosine A_2A_ receptor antagonists prevent the increase in striatal glutamate levels induced by glutamate uptake inhibitors. *Journal of Neurochemistry*.

[93] Pinto-Duarte A, Coelho JE, Cunha RA, Ribeiro JA, Sebastião AM (2005). Adenosine A_2A_ receptors control the extracellular levels of adenosine through modulation of nucleoside transporters activity in the rat hippocampus. *Journal of Neurochemistry*.

[94] Matos M, Augusto E, Santos-Rodrigues AD (2012). Adenosine A_2A_ receptors modulate glutamate uptake in cultured astrocytes and gliosomes. *GLIA*.

[95] Matos M, Augusto E, Agostinho P, Cunha RA, Chen JF (2013). Antagonistic interaction between adenosine A_2A_ receptors and Na^+^/K^+^-ATPase-*α*2 controlling glutamate uptake in astrocytes. *Journal of Neuroscience*.

[96] Coelho JE, Rebola N, Fragata I, Ribeiro JA, de Mendonça A, Cunha RA (2006). Hypoxia-induced desensitization and internalization of adenosine A_1_ receptors in the rat hippocampus. *Neuroscience*.

[97] Lopes LV, Cunha RA, Ribeiro JA (1999). Cross talk between A_1_ and A_2A_ adenosine receptors in the hippocampus and cortex of young adult and old rats. *Journal of Neurophysiology*.

[98] Ciruela F, Casadó V, Rodrigues RJ (2006). Presynaptic control of striatal glutamatergic neurotransmission by adenosine A_1_-A_2A_ receptor heteromers. *Journal of Neuroscience*.

[99] Dias RB, Ribeiro JA, Sebastião AM (2012). Enhancement of AMPA currents and GluR1 membrane expression through PKA-coupled adenosine A_2A_ receptors. *Hippocampus*.

[100] Rebola N, Lujan R, Cunha RA, Mulle C (2008). Adenosine A_2A_ receptors are essential for long-term potentiation of NMDA-EPSCs at hippocampal mossy fiber synapses. *Neuron*.

[101] Azdad K, Gall D, Woods AS, Ledent C, Ferré S, Schiffmann SN (2009). Dopamine D_2_ and adenosine A_2A_ receptors regulate NMDA-mediated excitation in accumbens neurons through A_2A_-D_2_ receptor heteromerization. *Neuropsychopharmacology*.

[102] Martire A, Ferrante A, Potenza RL (2010). Remodeling of striatal NMDA receptors by chronic A_2A_ receptor blockade in Huntington's disease mice. *Neurobiology of Disease*.

[103] Alagarsamy S, Rouse ST, Gereau RW, Heinemann SF, Smith Y, Conn PJ (1999). Activation of N-methyl-D-aspartate receptors reverses desensitization of metabotropic glutamate receptor, mGluR5, in native and recombinant systems. *Annals of the New York Academy of Sciences*.

[104] Tebano MT, Martire A, Rebola N (2005). Adenosine A_2A_ receptors and metabotropic glutamate 5 receptors are co-localized and functionally interact in the hippocampus: a possible key mechanism in the modulation of N-methyl-D-aspartate effects. *Journal of Neurochemistry*.

[105] Moroni F, Chiarugi A (2009). Post-ischemic brain damage: targeting PARP-1 within the ischemic neurovascular units as a realistic avenue to stroke treatment. *The FEBS Journal*.

[106] Trincavelli ML, Melani A, Guidi S (2008). Regulation of A_2A_ adenosine receptor expression and functioning following permanent focal ischemia in rat brain. *Journal of Neurochemistry*.

[107] Popoli P, Pintor A, Domenici MR (2002). Blockade of striatal adenosine A_2A_ receptor reduces, through a presynaptic mechanism, quinolinic acid-induced excitotoxicity: possible relevance to neuroprotective interventions in neurodegenerative diseases of the striatum. *Journal of Neuroscience*.

[108] Schwartz-Bloom RD, Sah R (2001). *γ*-aminobutyric acid_A_ neurotransmission and cerebral ischemia. *Journal of Neurochemistry*.

[109] O’Regan MH, Simpson RE, Perkins LM, Phillis JW (1992). Adenosine receptor agonists inhibit the release of *γ*-aminobutyric acid (GABA) from the ischemic rat cerebral cortex. *Brain Research*.

[110] Saransaari P, Oja SS (2005). GABA release modified by adenosine receptors in mouse hippocampal slices under normal and ischemic conditions. *Neurochemical Research*.

[111] Cristóvão-Ferreira S, Vaz SH, Ribeiro JA, Sebastião AM (2009). Adenosine A_2A_ receptors enhance GABA transport into nerve terminals by restraining PKC inhibition of GAT-1. *Journal of Neurochemistry*.

[112] Stone TW, Behan WMH (2007). Interleukin-1*β* but not tumor necrosis factor-*α* potentiates neuronal damage by quinolinic acid: protection by an adenosine A_2A_ receptor antagonist. *Journal of Neuroscience Research*.

[113] Irving EA, Barone FC, Reith AD, Hadingham SJ, Parsons AA (2000). Differential activation of MAPK/ERK and p38/SAPK in neurones and glia following focal cerebral ischaemia in the rat. *Molecular Brain Research*.

[114] Wu DC, Ye W, Che XM, Yang GY (2000). Activation of mitogen-activated protein kinases after permanent cerebral artery occlusion in mouse brain. *Journal of Cerebral Blood Flow and Metabolism*.

[115] Takagi Y, Nozaki K, Sugino T, Hattori I, Hashimoto N (2000). Phosphorylation of c-Jun NH_2_-terminal kinase and p38 mitogen-activated protein kinase after transient forebrain ischemia in mice. *Neuroscience Letters*.

[116] Piao CS, Kim JB, Han PL, Lee JK (2003). Administration of the p38 MAPK inhibitor SB203580 affords brain protection with a wide therapeutic window against focal ischemic insult. *Journal of Neuroscience Research*.

[117] Bruce-Keller AJ (1999). Microglial-neuronal interactions in synaptic damage and recovery. *Journal of Neuroscience Research*.

[118] Barone FC, Irving EA, Ray AM (2001). Inhibition of p38 mitogen-activated protein kinase provides neuroprotection in cerebral focal ischemia. *Medicinal Research Reviews*.

[119] Rebola N, Simões AP, Canas PM (2011). Adenosine A_2A_ receptors control neuroinflammation and consequent hippocampal neuronal dysfunction. *Journal of Neurochemistry*.

[120] Orr AG, Orr AL, Li X, Gross RE, Traynelis SF (2009). Adenosine A_2A_ receptor mediates microglial process retraction. *Nature Neuroscience*.

[121] Gomes C, Ferreira R, George J (2013). Activation of microglial cells triggers a release of brain-derived neurotrophic factor (BDNF) inducing their proliferation in an adenosine A_2A_ receptor-dependent manner: A_2A_ receptor blockade prevents BDNF release and proliferation of microglia. *Journal of Neuroinflammation*.

[122] Saura J, Angulo E, Ejarque A (2005). Adenosine A_2A_ receptor stimulation potentiates nitric oxide release by activated microglia. *Journal of Neurochemistry*.

[123] Melani A, Corti F, Vannucchi MG, Nosi D, Giovannini MG, Pedata F (2011). Role of A_2A_ receptors on modulation of oligodendroglia in cerebral ischemia. *Shock*.

[124] Howe CL, Bieber AJ, Warrington AE, Pease LR, Rodriguez M (2004). Antiapoptotic signaling by a remyelination-promoting human antimyelin antibody. *Neurobiology of Disease*.

[125] Jurewicz A, Matysiak M, Andrzejak S, Selmaj K (2006). TRAIL-induced death of human adult oligodendrocytes is mediated by JNK pathway. *GLIA*.

[126] Bonetti B, Stegagno C, Cannella B, Rizzuto N, Moretto G, Raine CS (1999). Activation of NF-*κ*B and c-jun transcription factors in multiple sclerosis lesions: implications for oligodendrocyte pathology. *The American Journal of Pathology*.

[127] Hirt L, Badaut J, Thevenet J (2004). D-JNKI1, a cell-penetrating c-Jun-N-terminal kinase inhibitor, protects against cell death in severe cerebral ischemia. *Stroke*.

[128] Borsellol T, Clarkel PGH, Hirt L (2003). A peptide inhibitor of c-Jun N-terminal kinase protects against excitotoxicity and cerebral ischemia. *Nature Medicine*.

[129] Kuan CY, Whitmarsh AJ, Yang DD (2003). A critical role of neural-specific JNK3 for ischemic apoptosis. *Proceedings of the National Academy of Sciences of the United States of America*.

[130] Gelderblom M, Eminel S, Herdegen T, Waetzig V (2004). c-Jun N-terminal kinases (JNKs) and the cytoskeleton—functions beyond neurodegeneration. *International Journal of Developmental Neuroscience*.

[131] Kitada M, Rowitch DH (2006). Transcription factor co-expression patterns indicate heterogeneity of oligodendroglial subpopulations in adult spinal cord. *GLIA*.

[132] Coppi E, Cellai L, Maraula G, Pugliese AM, Pedata F (2013). Adenosine A_2A_ receptors inhibit delayed rectifier potassium currents and cell differentiation in primary purified oligodendrocyte cultures. *Neuropharmacology*.

[133] Shrager P, Novakovic SD (1995). Control of myelination, axonal growth, and synapse formation in spinal cord explants by ion channels and electrical activity. *Developmental Brain Research*.

[134] Attali B, Wang N, Kolot A, Sobko A, Cherepanov V, Soliven B (1997). Characterization of delayed rectifier Kv channels in oligodendrocytes and progenitor cells. *Journal of Neuroscience*.

[135] Matute C, Sánchez-Gómez MV, Martínez-Millán L, Miledi R (1997). Glutamate receptor-mediated toxicity in optic nerve oligodendrocytes. *Proceedings of the National Academy of Sciences of the United States of America*.

[136] Matute C, Alberdi E, Ibarretxe G, Sánchez-Gómez MV (2002). Excitotoxicity in glial cells. *European Journal of Pharmacology*.

[137] Mcdonald JW, Althomsons SP, Hyrc KL, Choi DW, Goldberg MP (1998). Oligodendrocytes from forebrain are highly vulnerable to AMPA/kainate receptor-mediated excitotoxicity. *Nature Medicine*.

[138] Back SA (2006). Perinatal white matter injury: the changing spectrum of pathology and emerging insights into pathogenetic mechanisms. *Mental Retardation and Developmental Disabilities Research Reviews*.

[139] Kawasaki H, Morooka T, Shimohama S (1997). Activation and involvement of p38 mitogen-activated protein kinase in glutamate-induced apoptosis in rat cerebellar granule cells. *Journal of Biological Chemistry*.

[140] Tikka TM, Koistinaho JE (2001). Minocycline provides neuroprotection against N-methyl-D-aspartate neurotoxicity by inhibiting microglia. *Journal of Immunology*.

[141] Lewis AJ, Manning AM (1999). New targets for anti-inflammatory drugs. *Current Opinion in Chemical Biology*.

[142] Dai SS, Zhou YG, Li W (2010). Local glutamate level dictates adenosine A_2A_ receptor regulation of neuroinflammation and traumatic brain injury. *Journal of Neuroscience*.

[143] Ådén U, Halldner L, Lagercrantz H, Dalmau I, Ledent C, Fredholm BB (2003). Aggravated brain damage after hypoxic ischemia in immature adenosine A_2A_ knockout mice. *Stroke*.

[144] Castillo A, Tolón MR, Fernández-Ruiz J, Romero J, Martinez-Orgado J (2010). The neuroprotective effect of cannabidiol in an *in vitro* model of newborn hypoxic-ischemic brain damage in mice is mediated by CB_2_ and adenosine receptors. *Neurobiology of Disease*.

[145] Duan W, Gui L, Zhou Z (2009). Adenosine A_2A_ receptor deficiency exacerbates white matter lesions and cognitive deficits induced by chronic cerebral hypoperfusion in mice. *Journal of the Neurological Sciences*.

[146] Phillis JW (2004). Adenosine and adenine nucleotides as regulators of cerebral blood flow: roles of acidosis, cell swelling, and KATP channels. *Critical Reviews in Neurobiology*.

[147] Schindler CW, Karcz-Kubicha M, Thorndike EB (2004). Lack of adenosine A_1_ and dopamine D_2_ receptor-mediated modulation of the cardiovascular effects of the adenosine A_2A_ receptor agonist CGS 21680. *European Journal of Pharmacology*.

[148] Schindler CW, Karcz-Kubicha M, Thorndike EB (2005). Role of central and peripheral adenosine receptors in the cardiovascular responses to intraperitoneal injections of adenosine A_1_ and A_2A_ subtype receptor agonists. *British Journal of Pharmacology*.

[149] Wardas J, Konieczny J, Pietraszek M (2003). Influence of CGS 21680, a selective adenosine A_2A_ agonist, on the phencyclidine-induced sensorimotor gating deficit and motor behaviour in rats. *Psychopharmacology*.

[150] Mayne M, Fotheringham J, Yan HJ (2001). Adenosine A_2A_ receptor activation reduces proinflammatory events and decreases cell death following intracerebral hemorrhage. *Annals of Neurology*.

[151] Sebastião AM, Ribeiro JA (2009). Triggering neurotrophic factor actions through adenosine A_2A_ receptor activation: implications for neuroprotection. *British Journal of Pharmacology*.

[152] Heese K, Fiebich BL, Bauer J, Otten U (1997). Nerve growth factor (NGF) expression in rat microglia is induced by adenosine A_2A_-receptors. *Neuroscience Letters*.

[153] Tebano MT, Martire A, Potenza RL (2008). Adenosine A_2A_ receptors are required for normal BDNF levels and BDNF-induced potentiation of synaptic transmission in the mouse hippocampus. *Journal of Neurochemistry*.

[154] Jeon SJ, Rhee SY, Ryu JH (2011). Activation of adenosine A_2A_ receptor up-regulates BDNF expression in rat primary cortical neurons. *Neurochemical Research*.

[155] Gomes CARV, Vaz SH, Ribeiro JA, Sebastião AM (2006). Glial cell line-derived neurotrophic factor (GDNF) enhances dopamine release from striatal nerve endings in an adenosine A_2A_ receptor-dependent manner. *Brain Research*.

[156] Jerónimo-Santos A, Batalha VL, Müller CE (2014). Impact of in vivo chronic blockade of adenosine A_2A_ receptors on the BDNF-mediated facilitation of LTP. *Neuropharmacology*.

[157] Jones PA, Smith RA, Stone TW (1998). Protection against hippocampal kainate excitotoxicity by intracerebral administration of an adenosine A_2A_ receptor antagonist. *Brain Research*.

[158] Paterniti I, Melani A, Cipriani S (2011). Selective adenosine A_2A_ receptor agonists and antagonists protect against spinal cord injury through peripheral and central effects. *Journal of Neuroinflammation*.

[159] Macrez R, Ali C, Toutirais O (2011). Stroke and the immune system: from pathophysiology to new therapeutic strategies. *The Lancet Neurology*.

[160] Haskó G, Linden J, Cronstein B, Pacher P (2008). Adenosine receptors: therapeutic aspects for inflammatory and immune diseases. *Nature Reviews Drug Discovery*.

[161] Antonioli L, Csóka B, Fornai M (2014). Adenosine and inflammation: what's new on the horizon?. *Drug Discovery Today*.

[162] Gebicke-Haerter PJ, Christoffel F, Timmer J, Northoff H, Berger M, Van Calker D (1996). Both adenosine A_1_- and A_2_-receptors are required to stimulate microglial proliferation. *Neurochemistry International*.

[163] Buttini M, Appel K, Sauter A, Gebicke-Haerter P-J, Boddeke HWGM (1996). Expression of tumor necrosis factor alpha after focal cerebral ischaemia in the rat. *Neuroscience*.

[164] Iadecola C, Anrather J (2011). Stroke research at a crossroad: asking the brain for directions. *Nature Neuroscience*.

[165] Gelderblom M, Leypoldt F, Steinbach K (2009). Temporal and spatial dynamics of cerebral immune cell accumulation in stroke. *Stroke*.

[166] Sandoval KE, Witt KA (2008). Blood-brain barrier tight junction permeability and ischemic stroke. *Neurobiology of Disease*.

[167] Kuroiwa T, Ting P, Martinez H, Klatzo I (1985). The biphasic opening of the blood-brain barrier to proteins following temporary middle cerebral artery occlusion. *Acta Neuropathologica*.

[168] Akopov SE, Simonian NA, Grigorian GS (1996). Dynamics of polymorphonuclear leukocyte accumulation in acute cerebral infarction and their correlation with brain tissue damage. *Stroke*.

[169] Zhang RL, Chopp M, Chen H, Garcia JH (1994). Temporal profile of ischemic tissue damage, neutrophil response, and vascular plugging following permanent and transient (2H) middle cerebral artery occlusion in the rat. *Journal of the Neurological Sciences*.

[170] Cassada DC, Tribble CG, Young JS (2002). Adenosine A_2A_ analogue improves neurologic outcome after spinal cord trauma in the rabbit. *Journal of Trauma-Injury Infection & Critical Care*.

[171] Genovese T, Melani A, Esposito E (2009). The selective adenosine A_2A_ receptor agonist CGS 21680 reduces JNK MAPK activation in oligodendrocytes in injured spinal cord. *Shock*.

[172] Genovese T, Melani A, Esposito E (2010). Selective adenosine A_2A_ receptor agonists reduce the apoptosis in an experimental model of spinal cord trauma. *Journal of Biological Regulators and Homeostatic Agents*.

[173] Yao S, Li Z, Huang Q (2012). Genetic inactivation of the adenosine A_2A_ receptor exacerbates brain damage in mice with experimental autoimmune encephalomyelitis. *Journal of Neurochemistry*.

[174] Li Y, Oskouian RJ, Day Y-J (2006). Mouse spinal cord compression injury is reduced by either activation of the adenosine A_2A_ receptor on bone marrow-derived cells or deletion of the A_2A_ receptor on non-bone marrow-derived cells. *Neuroscience*.

[175] Haskó G, Kuhel DG, Chen J (2000). Adenosine inhibits IL-12 and TNF-*α* production via adenosine A_2A_ receptor-dependent and independent mechanism. *The FASEB Journal*.

[176] Lappas CM, Day YJ, Marshall MA, Engelhard VH, Linden J (2006). Adenosine A_2A_ receptor activation reduces hepatic ischemia reperfusion injury by inhibiting CD1d-dependent NKT cell activation. *Journal of Experimental Medicine*.

[177] Sitkovsky MV (2003). Use of the A_2A_ adenosine receptor as a physiological immunosuppressor and to engineer inflammation in vivo. *Biochemical Pharmacology*.

[178] Sitkovsky MV, Lukashev D, Apasov S (2004). Physiological control of immune response and inflammatory tissue damage by hypoxia-inducible factors and adenosine A_2A_ receptors. *Annual Review of Immunology*.

[179] Odashima M, Bamias G, Rivera-Nieves J (2005). Activation of A_2A_ adenosine receptor attenuates intestinal inflammation in animal models of inflammatory bowel disease. *Gastroenterology*.

[180] Choukèr A, Thiel M, Lukashev D (2008). Critical role of hypoxia and A_2A_ adenosine receptors in liver tissue-protecting physiological anti-inflammatory pathway. *Molecular Medicine*.

[181] Day YJ, Marshall MA, Huang L, McDuffie MJ, Okusa MD, Linden J (2004). Protection from ischemic liver injury by activation of A_2A_ adenosine receptors during reperfusion: inhibition of chemokine induction. *The American Journal of Physiology—Gastrointestinal and Liver Physiology*.

[182] Mazzon E, Esposito E, Impellizzeri D (2011). CGS 21680, an Agonist of the Adenosine (A_2A_) receptor, reduces progression of murine type II collagen-induced arthritis. *Journal of Rheumatology*.

[183] Impellizzeri D, di Paola R, Esposito E (2011). CGS 21680, an agonist of the adenosine (A_2A_) receptor, decreases acute lung inflammation. *European Journal of Pharmacology*.

[184] Di Paola R, Melani A, Esposito E (2010). Adenosine A_2A_ receptor-selective stimulation reduces signaling pathways involved in the development of intestine ischemia and reperfusion injury. *Shock*.

[185] Xu J, Guo S, Jia Z, Ma S, Li Z, Xue R (2013). Additive effect of prostaglandin E_2_ and adenosine in mouse experimental autoimmune encephalomyelitis. *Prostaglandins and Other Lipid Mediators*.

[186] Diógenes MJ, Fernandes CC, Sebastião AM, Ribeiro JA (2004). Activation of adenosine A_2A_ receptor facilitates brain-derived neurotrophic factor modulation of synaptic transmission in hippocampal slices. *The Journal of Neuroscience*.

[187] Diógenes MJ, Assaife-Lopes N, Pinto-Duarte A, Ribeiro JA, Sebastião AM (2007). Influence of age on BDNF modulation of hippocampal synaptic transmission: interplay with adenosine A_2A_ receptors. *Hippocampus*.

[188] Ekdahl CT, Claasen J, Bonde S, Kokaia Z, Lindvall O (2003). Inflammation is detrimental for neurogenesis in adult brain. *Proceedings of the National Academy of Sciences of the United States of America*.

[189] Yu L, Huang Z, Mariani JF, Wang Y, Moskowitz M, Chen J (2004). Selective inactivation or reconstitution of adenosine A_2A_ receptors in bone marrow cells reveals their significant contribution to the development of ischemic brain injury. *Nature Medicine*.

[190] Mostofsky E, Schlaug G, Mukamal KJ, Rosamond WD, Mittleman MA (2010). Coffee and acute ischemic stroke onset: the stroke onset study. *Neurology*.

[191] Smits P, Pieters G, Thien T (1986). The role of epinephrine in the circulatory effects of coffee. *Clinical Pharmacology and Therapeutics*.

[192] Nurminen M-L, Niittynen L, Korpela R, Vapaatalo H (1999). Coffee, caffeine and blood pressure: a critical review. *European Journal of Clinical Nutrition*.

[193] Mahmud A, Feely J (2001). Acute effect of caffeine on arterial stiffness and aortic pressure waveform. *Hypertension*.

[194] Papamichael CM, Aznaouridis KA, Karatzis EN (2005). Effect of coffee on endothelial function in healthy subjects: the role of caffeine. *Clinical Science*.

[195] Rivera-Oliver M, Díaz-Ríos M (2014). Using caffeine and other adenosine receptor antagonists and agonists as therapeutic tools against neurodegenerative diseases: a review. *Life Sciences*.

[196] Bøhn SK, Ward NC, Hodgson JM, Croft KD (2012). Effects of tea and coffee on cardiovascular disease risk. *Food & function*.

[197] Larsson SC, Orsini N (2011). Coffee consumption and risk of stroke: a dose-response meta-analysis of prospective studies. *American Journal of Epidemiology*.

[198] Grobbee DE, Rimm EB, Giovannucci E, Colditz G, Stampfer M, Willett W (1990). Coffee, caffeine, and cardiovascular disease in men. *New England Journal of Medicine*.

[199] Lopez-Garcia E, Rodriguez-Artalejo F, Rexrode KM, Logroscino G, Hu FB, Van Dam RM (2009). Coffee consumption and risk of stroke in women. *Circulation*.

[200] Larsson SC, Virtamo J, Wolk A (2011). Coffee consumption and risk of stroke in women. *Stroke*.

[201] Montagnana M, Favaloro EJ, Lippi G (2012). Coffee intake and cardiovascular disease: virtue does not take center stage. *Seminars in Thrombosis & Hemostasis*.

[202] Cristalli G, Müller CE, Volpini R (2009). Recent developments in Adenosine A_2A_ receptor ligands. *Handbook of Experimental Pharmacology*.

[203] Jacobson KA (2013). Structure-based approaches to ligands for G-protein-coupled adenosine and P2Y receptors, from small molecules to nanoconjugates. *Journal of Medicinal Chemistry*.

